# Anticancer Molecular Mechanisms of Phytosterols: An Updated Review on Clinical Trials

**DOI:** 10.1002/fsn3.71505

**Published:** 2026-02-03

**Authors:** Muhammad Shahbaz, Ushna Momal, Asfa Perween, Hammad Naeem, Muzzamal Hussain, Muhammad Imran, Gamal A. Mohamed, Sabrin R. M. Ibrahim, Suliman A. Alsagaby, Waleed Al Abdulmonem, Entessar Al Jbawi, Mohamed A. Abdelgawad, Samy Selim, Soad K. Al Jaouni, Hagar M. Mohamed

**Affiliations:** ^1^ Department of Food Science and Technology Muhammad Nawaz Shareef University of Agriculture Multan Pakistan; ^2^ Department of Human Nutrition and Dietetics Muhammad Nawaz Shareef University of Agriculture Multan Pakistan; ^3^ Department of Food Science Government College University Faisalabad Faisalabad Pakistan; ^4^ Department of Food Science and Technology University of Narowal Narowal Pakistan; ^5^ Department of Natural Products and Alternative Medicine, Faculty of Pharmacy King Abdulaziz University Jeddah Saudi Arabia; ^6^ Department of Chemistry Preparatory Year Program, Batterjee Medical College Jeddah Saudi Arabia; ^7^ Department of Medical Laboratory Sciences, College of Applied Medical Sciences Majmaah University AL‐Majmaah Saudi Arabia; ^8^ Department of Pathology, College of Medicine Qassim University Buraidah Saudi Arabia; ^9^ Sugar Beet Research Department Crop Research Administration, General Commission for Scientific Agricultural Research (GCSAR) Damascus Syria; ^10^ Department of Pharmaceutical Chemistry, College of Pharmacy Jouf University Sakaka Saudi Arabia; ^11^ Department of Clinical Laboratory Sciences, College of Applied Medical Sciences Jouf University Sakaka Saudi Arabia; ^12^ Department of Hematology/Oncology, Chair of Prophetic Medicine Application, Faculty of Medicine King Abdulaziz University and Hospital Jeddah Saudi Arabia; ^13^ Department of Medical Laboratory Analysis, College of Medical and Health Sciences Liwa University Abu Dhabi UAE; ^14^ Department of Applied Medical Chemistry, Medical Research Institute Alexandria University Alexandria Egypt

**Keywords:** adjunctive component, anti‐inflammatory, cell growth, DNA damage, metastasis

## Abstract

Phytosterols, a form of naturally occurring substance structurally related to cholesterol, have been getting considerable interest due to their possible anticancer property. They have multifactorial modes of action such as antioxidant, anti‐inflammatory, and apoptotic, which render them useful in the prevention and treatment of prostate, breast, colon, bladder, and skin cancer. Phytosterol prevents cancer development by scavenging reactive oxidative species (ROS) and boosting antioxidant enzymes, thus inhibiting DNA damage and cell mutations that cause cancerous development. They also regulate important signal transduction processes such as NF‐kB, PI3K/Akt, and MAPK/ERK that drive cell growth, survival, and metastasis. Phytosterols induce apoptosis, block the cell cycle, and abrogate the invasion and metastasis of cancer cells, offering a multi‐manifestation treatment of cancer. Nevertheless, their clinical use is limited due to factors like low bioavailability, which can be overcome with research in nanotechnology and drug delivery schemes. However, based on preclinical and epidemiological studies, phytosterols can be used as a useful adjunctive component to cancer treatments. More studies are required to work out clinical testing and streamlined delivery to maximize their effectiveness in cancer treatment.

## Graphical abstract

Phytosterols, a form of naturally occurring substance structurally related to cholesterol, have been getting considerable interest due to their possible anticancer properties. Phytosterols induce apoptosis, block the cell cycle, and abrogate the invasion and metastasis of cancer cells, offering a multi‐manifestation treatment of cancer. Based on preclinical and epidemiological studies, phytosterols can be used as a useful adjunctive component to cancer treatments.

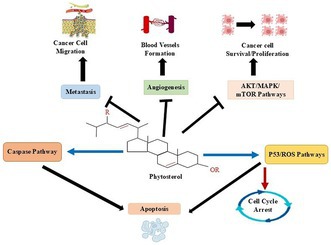

## 1 Introduction

Phytosterols (PSs) are naturally found plant‐based steroidal compounds, which constitute the most significant percentage of unsaponifiable lipid content in plants. They are structurally related to cholesterol and contain a steroid backbone having a hydroxyl group at C‐3 and an aliphatic chain at C‐17, unlike animal sterols. Over 250 phytosterols were found in plant life, with the most common types of phytosterols being 2‐sitosterol, campesterol, and stigmasterol. These bioactive molecules are found in the plant‐based foods, including vegetable oils, nuts, seeds, legumes, and whole grains, in high concentrations. Phytosterols have garnered growing scientific and clinical interest due to their diverse pharmacological effects, including cholesterol‐lowering, anti‐inflammatory, antioxidant, antidiabetic, and chemopreventive actions. Different sources of phytosterols are described in Table 1. Modulation of lipid metabolism, inhibition of cholesterol absorption, and improvement of cardiovascular health have led to their incorporation in functional foods and nutraceuticals (Salehi et al. 2021). Guggulsterone is a bioactive phytosterol derived from *Commiphora wightii*, which has strong anti‐cancer effects due to its ability to regulate various apoptotic and signaling pathways. It is a farnesoid X receptor (FXR) antagonist that regulates bile acid and cholesterol homeostasis and causes apoptosis and proliferation inhibition in a wide range of different types of cancer, such as pancreatic, hepatic, colorectal, breast, lung cancer, and many others. Mechanistically, guggulsterone can regulate the activity of such key molecular targets as NF‐kB, STAT3, PI3K/Akt, and caspases and induce mitochondrial dysfunction, inhibition of anti‐apoptotic factors, and reversal of multidrug resistance. In a systematic review and meta‐analysis, it was shown that it serves as a strong time‐ and dose‐dependent pro‐apoptotic and has potential as a natural anticancer agent to be explored further in preclinical and clinical studies (Gupta et al. 2023). Phytochemicals are classified into several compounds and have been proposed to be effective in treating and preventing diseases because they are antioxidant agents and have anti‐inflammatory properties (Ahmad et al. 2019). This therapeutic property of theirs is based on the fact that they alter multiple cellular processes that are involved in carcinogenesis by modulating them, representing a potentially effective form of chemoprevention and adjunctive cancer treatment (Sitati 2025). The review paper is intended to be a synthesis of the present‐day knowledge of the anticancer functionality of phytosterol and a part of a critical analysis of the results of recent clinical trials. In addition to their immediate antineoplastic effects, phytosterols have the potential to moderate treatment resistance and reduce the adverse effects of conventional cancer medicine. As an example, prostate cancer, which is one of the leading causes of death in men, has the potential to trigger cell cycle arrest and apoptosis of tumor cells due to phytosterols, preventing tumor cell growth and suppressing angiogenesis (Iheagwam et al. 2025).

**TABLE 1:** Major sources, types, and health benefits of phytosterols.

Source	Major phytosterol types	Primary health benefits	References
Fruits (e.g., berries, citrus, apples)	β‐sitosterol, stigmasterol	Enhances endothelial function, reduces oxidative stress, supports cardiovascular and metabolic health	Fałczyńska et al. (2025)
Fruit Seed Oils (e.g., grape, strawberry, pomegranate)	Stigmasterol, β‐sitosterol	Antioxidant, hepatoprotective, and anti‐inflammatory activity; promotes dermal and cardiovascular health	Rodríguez‐Blázquez et al. (2025)
Vegetable Oils (e.g., sunflower, rapeseed, soybean, palm)	β‐sitosterol, campesterol, stigmasterol	Improves lipid metabolism, reduces LDL cholesterol, and exhibits antioxidant and anti‐inflammatory effects	Tian et al. (2023)
Whole Grains (e.g., wheat, oats, barley)	Campesterol, β‐sitosterol, ferulic ester conjugates	Improves gut microbiota composition, reduces CVD risk, exhibits anticancer potential through fiber–phytosterol synergy	Hafez‐Ghoran et al. (2025)
Nuts and Seeds (e.g., almonds, flaxseed, pumpkin, sesame)	β‐sitosterol, avenasterol, campesterol	Cardioprotective; lowers plasma cholesterol; modulates oxidative stress; supports endothelial function	Dodevska et al. (2022)

**FIGURE 1:**
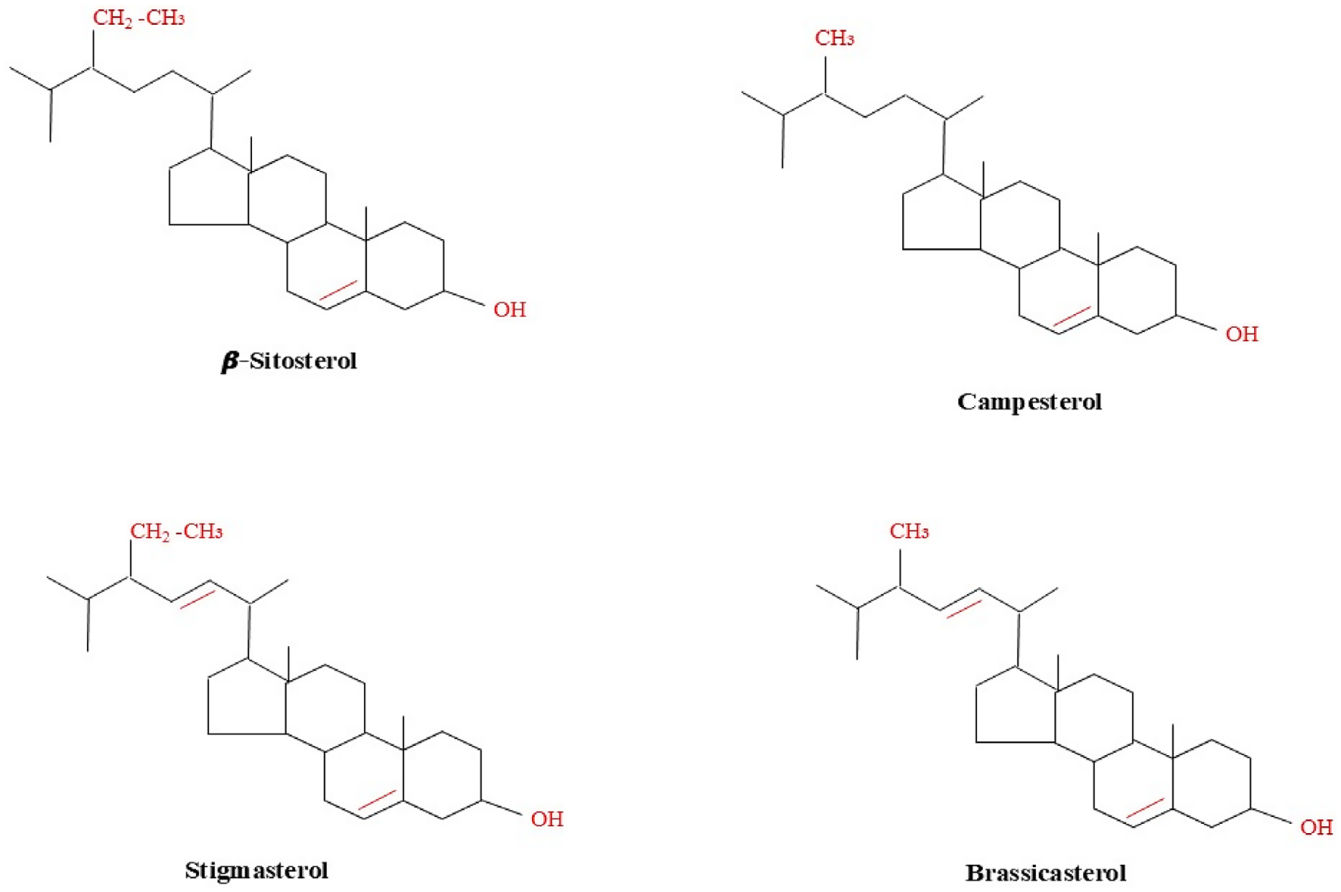
Chemical structures of phytosterols.

Similarly, phytosterols have potential in the prevention of breast cancer, delivering estrogen receptor signaling and the development of apoptotic mechanisms, which is a natural pharmacological intervention (Pandey et al. 2021). Moreover, the growing body of evidence suggests that a wide variety of natural compounds have the potential to be used successfully to target cancer cells but not harm healthy cells and be an effective addition to the existing cancer treatment methods (Krist 2020). The ability to target is critical in particular, due to the challenges of resistance development and systemic toxicity associated with the conventional chemotherapeutic compounds. The particular review of the impact of specific phytosterols (stigmasterol) proves their special anti‐tumorigenic effects that promote apoptosis, inhibit proliferation, metastasis, and invasion, and induce autophagy in cancer cells. Stigmasterol was shown to influence primary intracellular signaling pathways, such as Akt/mTOR and JAK/STAT, in both ovarian and gastric cancers, which also supports the high potential of its therapeutic application (Nunes et al. 2022). The antiproliferative and angiogenesis‐disruptive effects of stigmasterol have been discovered in human cholangiocarcinoma by inhibiting the tumor necrosis factor‐alpha and vascular endothelial growth factor receptor‐2 signatures. Other postulated mechanisms are the anti‐inflammatory and antioxidant activity of stigmasterol, which appear to explain the anticancer effect of the compound in minimizing cell damage and immune regulation (Sitati 2025). Multifactorial functions are also attributed to stigmasterol, including the regulation of the most significant signal pathways and molecular targets, which highlights the prospect of therapeutic use of this compound in various cancers. The anticancer effects of these other phytosterols, i.e., beta‐sitosterol and campesterol, are also strong and regulate cell cycle, apoptosis, and angiogenesis, as demonstrated in many pre‐clinical and clinical trials. These compounds have the potential to affect cell growth, differentiation, and apoptosis, which is the foundation of their chemopreventive and therapeutic activity against many cancers (Iheagwam et al. 2025). Figure 1 represents the structural differences in the side chains and the break of two bonds among the four most important phytosterols that define their different biological activities and physicochemical characteristics.

## 2 Bioavailability of Phytosterols

The bioavailability of phytosterols is highly vital in identifying their effectiveness as anti‐cancerous agents since it entails their absorption, distribution, metabolism, and excretion within the human body. This pharmacokinetic profile characterizes the concentration of active phytosterol metabolites reaching target tissues, and that is why it directly preconditions the clinical potential of such metabolites and requires special consideration in the case of clinical practice. In spite of their easy absorption, the absorption rates can be very different depending on other fats consumed along with them, as well as the form of the phytosterol (Manickam et al. 2020). In addition to it, genetic polymorphisms of transporter proteins and the presence of the individual microbiome in the gastrointestinal tract also play an important role in determining the bioavailability of phytosterol, which leads to the variability of the individual response to pharmacotherapy (Jones et al. 2019). However, it is important to appreciate the existence of such complicated bioavailability problems in designing effective phytosterol‐based interventions and in anticipating the physiological impact of these interventions on the different classes of patients. In this way, delivery systems need to be enhanced, and one should study the personalized absorption patterns to maximize the anticancer potential of phytosterols. This may be achieved by exploring new formulation methods, e.g., nanoemulsions or solid lipid nanoparticles, which enhance their solubility and permeability across biological membranes (Barkat et al. 2020). The most significant translational barrier of phytosterols is bioavailability. Although nanocarrier systems (e.g., liposomes, micelles, solid lipid nanoparticles) are identified by us as promising, future research needs to quantify the benefits of their pharmacokinetics with human absorption and tissue‐distribution data. There is an urgent need to conduct comparative trials to establish whether phytosterols delivered with nanoparticles can reach therapeutic levels in cancerous tissues. Such developments in delivery technology are aimed at overcoming their low aqueous solubility and high first‐pass metabolism to lead to greater concentrations of therapeutic levels within tumor sites. Nanotechnology‐based innovative approaches, e.g., lipid‐based nanoparticles, are also a promising method of enhancing the specificity and bioavailability of phytosterols, i.e., improving their anticancer and reducing off‐target effects (Mehta et al. 2025). Such nanotechnological advancements can overcome bad solubility, high melting point, and low absorption property, which restrict the therapeutic application of free phytosterols. Indeed, nanoparticles can enhance the solubility of phytochemicals, their stability, their bioavailability, protect degradation in the body at an early stage, and increase the half‐life of circulation (AbouSamra et al. 2023). This is comprised of the pharmaceutical conversion of phytosterols to nano‐sized drug delivery vehicles capable of greatly increasing bioavailability and therapeutic efficacy. As a drug delivery system, including liposomes, micelles, or nanogels, has served as the purpose of these nano‐drugs, they offer specific benefits to polyphenolic compounds, since they can address low bioavailability and low intrinsic solubility in vivo. This new approach is highly promising in improving the delivery of various phytochemicals, such as phytosterols, to cancer cells in an effort to multiply their therapeutic effect, but with reduced systemic toxicity (Liu et al. 2022).

## 3 Antioxidant Potential of Phytosterols

Phytosterols are types of plant substances, which have a cholesterol‐like structure, and the experimental research has demonstrated that they have an essential antioxidant potential (Jagtap et al. 2025). Such antioxidant effects make them a valuable source in a variety of therapeutic effects, also involving hypolipidemic, anti‐inflammatory, antiproliferative, and hypoglycemic effects (Dar et al. 2025). As far as decreasing oxidized Low‐Density Lipoprotein by phytosterols, results show that this substance can have an altering effect on LDL oxidation. It has been demonstrated that in plant sterols‐supplemented diets, there is a significant reduction of plasma LDL cholesterol. This lowering of LDL cholesterol is also accompanied by a lowering in oxidized LDL, which seems to indicate that phytosterols present an antioxidant effect (Mohamed 2025). Phytosterols, such as beta‐sitosterol, stigmasterol, and campesterol, have been shown to prevent changes in apoprotein structure and physicochemical characteristics of lipoproteins. Such modifications are normally related to copper‐induced lipoprotein lipid peroxidation. This inhibitory effect on lipid peroxidation of LDL is thought to be physiologically important (Olofinte et al. 2025). Phytosterols can also lower small dense LDL (sdLDL‐C), which are thought to be atherogenic (contribute to plaque in the arteries) more than the large LDL. This has been reported in both children and adults with hypercholesterolemia (Vezza et al. 2020). It is necessary to distinguish between phytosterols and their oxidation products. Although phytosterols have great advantages in antioxidizing effects, the oxidation product of phytosterols may also have pro‐oxidant effects, so the stability of phytosterols in foods is important. Phytosterols have been found to stimulate the production of important antioxidant enzymes, including superoxide dismutase, catalase, and glutathione. These enzymes are crucial in the neutralization of reactive oxygen species in the body (Singh et al. 2025). Superoxide Dismutase breaks down superoxide radicals into hydrogen peroxide and molecular oxygen. Catalase converts hydrogen peroxide into water and oxygen. Glutathione is an antioxidant that aids in the detoxification of harmful chemicals and the maintenance of redox state. Phytosterols actively prevent lipid peroxidation, a process that causes the formation of free radicals, which oxidize the lipids, forming damaged cells and toxic byproducts (Datkhayev et al. 2025). This suppression is achieved in many different ways, including the induction of Nrf2 and Nrf2/Heme Oxygenase‐1 Signaling Pathways (Tian et al. 2025). One of the major regulators of the antioxidant activities is the nuclear factor erythroid‐2‐related factor 2. Once activated, Nrf2 proceeds to the nucleus to activate the expression of numerous antioxidant and detoxifying enzymes, including HO‐1. This modulation provides financing to the inbuilt systems of the cell to overcome oxidative stress. Other than antioxidants, phytosterols are also seen to possess numerous occurrences of inhibiting the production of pro‐inflammatory cytokines, which further supports them in their protective quality against oxidative stress and inflammation (Awari et al. 2024).

**FIGURE 2:**
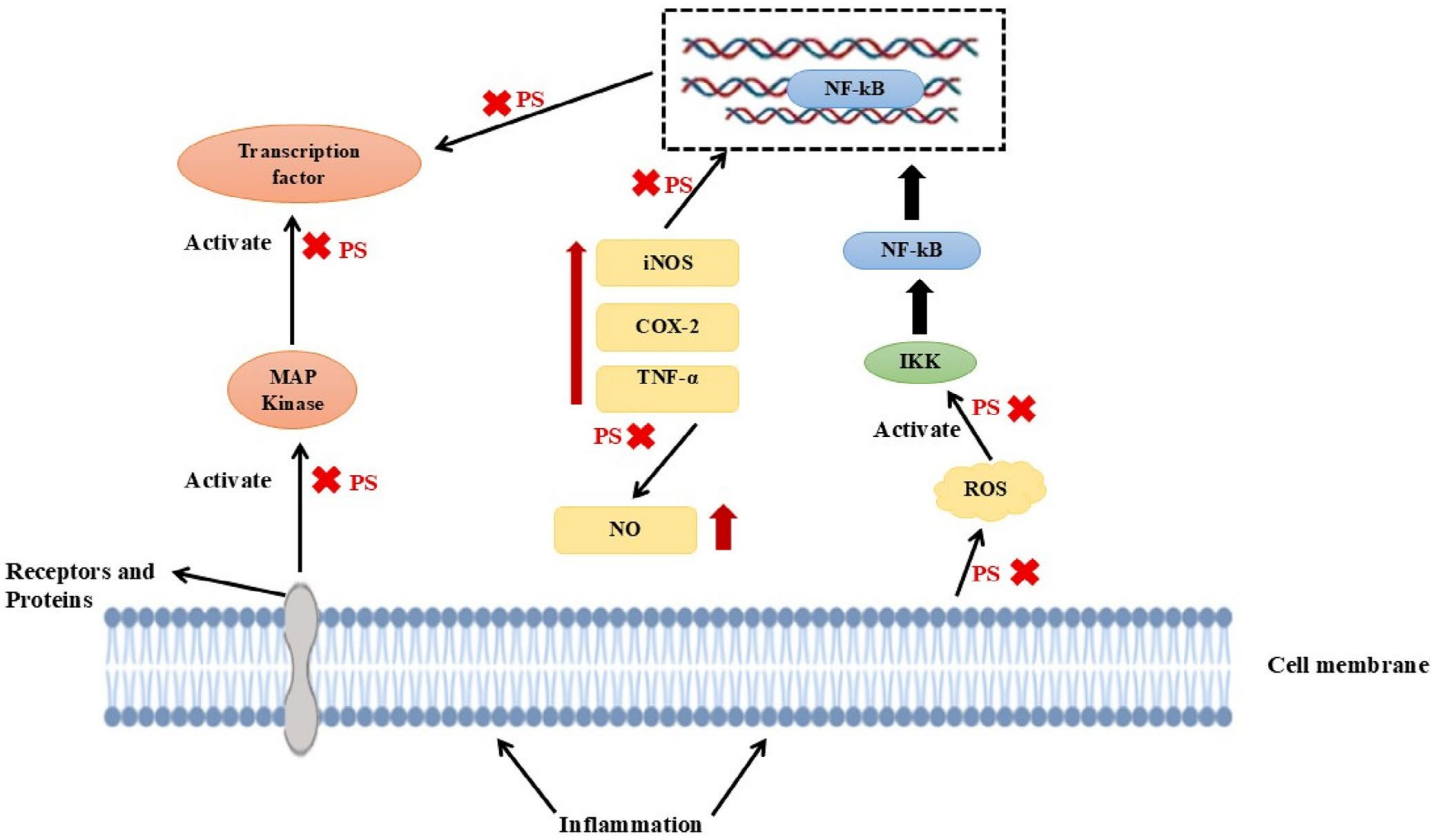
The mechanism related to the anti‐inflammatory activity of phytosterol.

A multidimensional solution, as such, implies the reasonable application of phytosterols to the oxidative stress‐induced cell damage and, therefore, to the maintenance of overall health (Shen et al. 2024). The study has implied that these compounds will help in the reduction of the small, dense LDL (sdLDL) particles, which are more atherogenic compared with the large LDL particles and a contributor to the risk of having a cardiovascular disease. Phytosterol supplementation has been proven to be linked with a significant reduction in the plasma levels of the small dense LDL (sdLDL). An experiment on patients with metabolic syndrome demonstrated a reduction of the levels of sdLDL in 2 months following the usage of phytosterols. Similarly, phytosterol‐fortified diets could lower the levels of sdLDL‐cholesterol in hypercholesterolemic children (Vezza et al. 2020). The Figure 2 also illustrates that phytosterols (PS) induce anti‐inflammatory effects by disrupting major signaling cascades by inflammatory stimuli (e.g., LPS, cytokines) in both the cell membrane and cytoplasm. PS inhibits or suppresses the generation of reactive oxygen species (ROS) and hence the inhibition of activation of upstream kinases such as MAPKs (ERK, p38, JNK) and IKK that phosphorylate IkB and cause degradation and nuclear translocation of NF‐kB. NF‐κB is inactive or less active with PS intervention, which lowers the expression of pro‐inflammatory genes, including TNF‐α, iNOS, COX‐2, and inhibits downstream effectors, including NO (Ning et al. 2024). PS also disrupts transcription factors that are downstream of MAPKs (e.g., AP‐1), thereby further inhibiting the release of inflammatory mediators. This type of mechanism is validated in many studies: β‐sitosterol was reported to reduce LPS‐induced IL‐6, COX‐2, TNF‐α, and iNOS expression by inhibiting the ERK/p38 and NF‐κB activation of BV2 microglial cells (Ong et al. 2022). In a similar manner, stigmasterol inhibited the activation of microglia and cytokine release through AMPK‐mediated inhibition of NF‐κB and NLRP3 signaling (AlMousa et al. 2025).

## 4 Clinical Trials

The clinical trials have been challenged to consistently show positive effects despite the promise of safe anticancer effects of phytosterols shown through preclinical and epidemiological studies. There are many reports on observation indicating that dietary phytosterols possibly have a negative relationship with the occurrence of certain cancers. A systematic meta‐analysis, as an example, offers evidence that high phytosterol intake has a negative association with the risk of cancer. It means that vegetable diets rich in compounds may help in cancer protective effects (Zio et al. 2024). Human clinical trials specifically designed to test the potential of phytosterols in cancer prevention are expensive to perform due to the size, nature, and complexities of conducting a large‐scale, randomized trial. As a result, in vitro (cell‐based) and preclinical, in vivo (in animal models) studies have provided much of our insight into the anticancer effect of phytosterols. The efficacy of the various types and forms of phytosterols in cancer prevention has been relatively assessed in limited studies that have evaluated interventional trials (Stellaard and Lütjohann 2025). This lack of consistency in human clinical data is probably due to different study designs, the diet of participants, and non‐standardized endpoints. As an example, small intervention trials of biomarker modulation do show but not all clinical outcomes are consistent. In solving this, future randomized controlled trials must incorporate standardized dosing, validated surrogate biomarkers, and long‐term follow‐up in order to determine clinical relevance. This shortage makes it hard to make conclusive findings based on clinical data alone. The outcomes of studies examining the relationships between dietary phytosterols and the risks of cancer have at times been inconsistent, even when studies are conducted. Such variability may be due to many factors, and it is difficult to draw causal correlations by relying solely on clinical trials. An important factor in this difference is the bioavailability of phytosterols. The absorption, distribution, metabolism, and excretion of phytosterols play a significant role in the efficacy of these compounds as anticancer agents in the human body (Ghanbari‐Gohari et al. 2025). Other factors that influence the extent of the active compound reaching a target tissue include dietary co‐ingestion, the presence of other fats, the chemical form of the phytosterol (esterified vs. free form), differences in genetic transporters of the protein, and the composition of an individual's gut microbiota. Phytosterols have a bioavailability of about 0.5%–2% (Ghaedi et al. 2020). This variability in absorption also makes it more difficult to conduct clinical trials, because concentrations cannot be easily maintained across a population. The unstandardized phytosterol preparations and variation of purity in studies inhibits the comparison of results. The preparation of international pharmacopeial standards and protocols of quantification which have been validated would enhance reproducibility and reduce the time taken in translating them to clinical use (Zhang, Yang, et al. 2025).

Future studies should aim at maximizing the delivery system and oxygen absorption profile in accordance to the individual. Various key barriers when carrying out clinical trials in cancer research on phytosterols emerge when discussing the feasibility challenges of conducting the clinical trials in cancer research. Trials and studies aiming to prevent cancer among humans by a large‐scale randomized trial are challenging, time‐consuming, and cost‐intensive in themselves. Thus, this makes their trials impossible in many natural compounds such as phytosterols despite their promise. The fact that cancer development takes a long latency period means that such studies take long and end up being expensive (Zio et al. 2024). As a result, the efficacy of different types and forms of phytosterols has not been directly evaluated with respect to cancer prevention or treatment in humans in a few interventional studies. It makes this clinical data a bit challenging to come up with a definite answer to their effectiveness in a clinical practice setting. It is important that clinical trials be conducted by well‐designed studies whereby confounding factors are taken care of. Dietary interventions on compounds like phytosterols are challenging because it is hard to be specific on consumption and is common to isolate the success of the specific compound and the general nutrition of the subjects, and not only the lifestyle habits (Miszczuk et al. 2024). Bioavailability of phytosterols has a significant influence on their effectiveness in clinical trials, and this is due to the mechanism of absorption, distribution, metabolism, and excretion of phytosterols in the body (Stellaard and Lütjohann 2025).

## 5 Clinical Evidence and Translational Outlook

Even though there is strong mechanistic evidence given by preclinical and in vitro results, few studies in humans have affirmed these effects. The available clinical trials have mixed results, which is mainly because of different phytosterol formulations, dosages, and study designs. As an example, the recent meta‐analyses indicate that tumor risks are decreased modestly with the intake of phytosterols but do not prove causation between the intake and the clinical outcome (Zio et al. 2024; Stellaard and Lütjohann 2025). Recent translational studies are starting to unravel the efficacy of phytosterols in a variety of cancers, with β‐sitosterol, the best‐investigated phytosterol, showing apoptosis and cell cycle arrest in breast, prostate, and pancreatic cancer by regulating PI3K/Akt, NF‐kB, and caspase pathways. These effects were validated by Zhang, Zhang, et al. (2025) in various tumor models and suggested using β‐sitosterol as a multi‐target therapeutic candidate with a high level of safety. In a multicenter in vivo study, β‐sitosterol (10–40 mg/kg/day, oral) was assessed in prostate and breast cancer xenograft models. Treatment had a potent effect in decreasing the tumor volume by 45%–60% with inhibition of PI3K/Akt/mTOR signaling. There was no hepatotoxicity or nephrotoxicity even at the maximum dose (Zhang, Zhang, et al. 2025).

Another phytosterol, namely stigmasterol, possesses extensive anticancer properties and was demonstrated to inhibit Akt/mTOR signaling and prevent metastasis in gastric, ovarian, and hepatic cancer models. The potential of stigmasterol at 200–400 mg/kg/day (oral, 4 weeks) in preventing and causing apoptosis in hepatic and ovarian carcinoma models was observed through down‐regulation of Bcl‐2 and activation of caspase‐9. Tumor inhibition was higher by 35% when doxorubicin was co‐administered without any increase in systemic toxicity. These results confirm that it may be used as an adjunctive treatment particularly in combination with conventional chemotherapeutics (Li et al. 2025). Dietary oils like flax and sunflower have been converted into campesterol and mixed phytosterol which have shown synergistic antioxidant and antiproliferative effects in oxidative stress‐induced cancers (Mubeen et al. 2025). These formulations can be used in a translational setting to promote redox balance of the system and to decrease tumor‐enhancing inflammation. A rat model under controlled conditions was fed on a 4RF21 diet that gave a 400 mg/kg/day combination of phytosterols after a duration of 6 weeks. The program reduced the colonic tumor multiplication by 52% through down‐regulation of COX‐2 and up‐regulation of p53 pathways (Yoon et al. 2025). A human pilot study (*n* = 48, colorectal and gastric cancer) used a 2 g/day mixed phytosterol supplement as administered to human subjects during 8 weeks of concomitant chemotherapy. The serum lipid peroxidation reduced by 23%, and the fatigue scores of the patients increased without adverse incidents (Akkol and Acıkara 2023). In addition, integrative studies show that phytosterols are not only acting on tumor cells but also on a tumor microenvironment. As an example, β‐sitosterol suppresses and reeducates immune control points and re‐plasma tumor‐linked macrophages into an antitumor surface (Monica et al. 2025).

## 6 Anticancer Perspectives

The phytosterols are also reported to stop the development of cancer, as they inhibit the unwanted cellular transformation and induce a premature cell death (apoptosis) of the diseased cells. It has been found in other types of cancer, including prostate cancer, in which phytosterols cause cell cycle arrest and cell death (apoptosis), suppressing the proliferation of tumor cells. It was found that phytosterols (stigmasterol, beta‐sitosterol, and campesterol) affected the proliferation, differentiation, and programmed cell death. One of them is to apply beta‐sitosterol that possesses pro‐apoptotic, anti‐proliferative, anti‐metastatic, anti‐invasive, and chemo‐sensitizing properties against tumor cells (Ahmad et al. 2019). The vital signaling networks are disrupted by phytosterols, which are often deregulated in cancer. Indicatively, stigmasterol has been disclosed to control pertinent intracellular signaling networks comprising Akt/mTOR and JAK/STAT in ovarian and gastric malignancies. They also act on reprogramming of metabolism and oxidative stress, among others. Phytosterols were also found to inhibit the development of new blood vessels that feed cancer (angiogenesis), and transfer of cancer cells to other body parts (metastasis) (Nunes et al. 2022). Stigmasterol is also another example that has been reported to affect the inhibition of angiogenesis of human cholangiocarcinoma. Phytosterol has also been shown to reduce primary and secondary tumor burden in pre‐clinical models and invariably reduce indicators of metastasis and angiogenesis in breast and colorectal cancer (Prieto and Hanafi 2023). Phytosterols can also be used to reduce resistance to the conventional treatment of cancer. Experiments have strongly suggested that phytosterols (e.g., beta‐sitosterol) possess a large number of anticancer effects in a wide range of cancers, including liver, cervix, stomach, breast, lung, pancreatic, and prostate, leukemia, multiple myeloma, melanoma, and fibrosarcoma (Shahzad et al. 2017).

**FIGURE 3:**
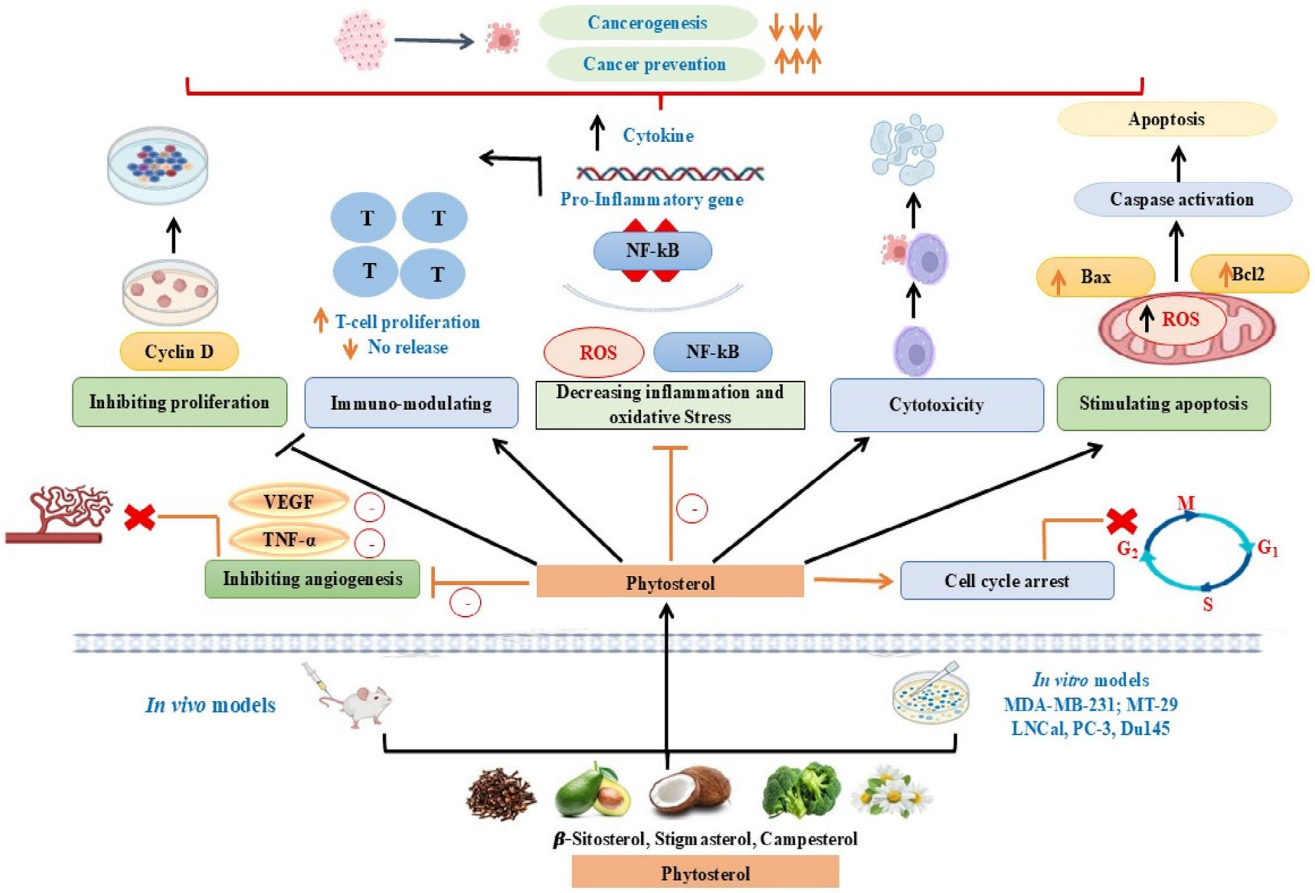
Anti‐cancer mechanism of phytosterol.

Phytosterols have been known to reduce cancers of the breast, prostate, lung, liver, stomach, and ovary, and their anticancer properties are summarized in the Table 2. Phytosterol consumption is even negatively correlated to the rates of occurrence and survival of various cancers. Phytosterol‐rich diets have been proposed to prevent the development of cancer by a fifth. Although there is excellent preclinical and epidemiological data, the translation of these findings consistently into clinical practice has proven problematic (Zio et al. 2024). Major concerns are the poor bioavailability of phytosterols, whose absorption, distribution, metabolism, and excretion in the human body could widely differ depending on factors such as dietary co‐consumption, the chemical form, and personal disparities. Low aqueous solubility and low targeting efficacy also affect their therapeutic efficacy and clinical application. Also, their inconsistent pharmacological activity and the absence of standardization can reduce their extensive clinical applicability, as has been mentioned in publications concerning phytosterols in benign prostatic hyperplasia. Future research should aim to maximize delivery mechanisms, such as nanotechnology‐based methodology (e.g., nanoemulsions, solid lipid nanoparticles, liposomes, micelles), to enhance solubility, permeability, and target‐specific delivery to the tumor region to boost its therapeutic potential and clinical utility (Zheng et al. 2019). The Figure 3 anticancer mechanism of phytosterol is a summary of the recent evidence on the role of phytosterols, including 2‐sitosterol, stigmasterol, and campesterol, in anti‐cancer activities by means of adjusting numerous pathways, including oxidative stress, inflammation, cell cycle, apoptosis, cytotoxicity, angiogenesis, and immunomodulation. As an example, β‐sitosterol has been reported to induce intrinsic and extrinsic apoptosis through upregulation of pro‐apoptotic BAX (or BAK), downregulation of BCL‐2 family members, caspase (−3/7/8/9) activity, mitochondrial outer membrane permeabilization, increase in reactive oxygen species (ROS), and inhibition of apoptotic inhibitors (IAPs) (Nandi et al. 2024).

**TABLE 2:** Anticancer properties of phytosterols.

Type of cancer	In vivo/in vitro	Cell line or model	Source	Mechanism	References
Cervical cancer	In vitro	CaSki cells, HeLa	β‐Sitosterol	Reduced PCNA (proliferation marker), lower expression of HPV E6, elevated p53; ultrastructural evidence of apoptosis	Cheng et al. (2015)
Brain cancer	In vivo In vitro	U87 xenografts; U87 glioma cells	β‐Sitosterol	In vitro: G2/M cell cycle arrest, apoptosis, downregulation of EMT markers, inhibited migration; Mechanism: prevents EGFR/MAPK signaling. In vivo: inhibited tumor growth	Xie et al. (2024)
Breast cancer	In vivo In vitro	MCF‐7 cells; MCF‐7 xenograft in mice	sweet potato phytosterols DLA/DL/DP; β‐Sitosterol derivatives (3β‐glucose sitosterol)	In vitro: causes apoptosis (Caspase‐3/9 upregulation), ERB, and caspase‐3 interaction. In vivo: inhibits tumor growth through gut microbiota regulation, SCFA release, downregulates PI3K/AKT/NF‐kB, activates caspases, reduces Ki67, VEGF, BCL‐2, BCL‐XL, and upregulates BAX	Han et al. (2021)
Ovarian cancer	In vitro	OV90 and ES2 cells	Stigmasterol	Apoptosis induced, ROS, mitochondrial/endoplasmic reticulum, calcium overload, and inhibited migration	Bae et al. (2020)
Colon cancer	In vitro	HCT‐15 cells	Novel phytosterol RinoxiaB	Induced apoptosis through mitochondrial damage, Cytochrome C release, caspase activation, BAX/Bcl‐2 modulation, p53/p21, cell cycle arrest (S and G2/M phase)	Gajendran et al. (2020)
Gastric cancer	In vitro	SGC‐7901	β‐Sitosterol	Inhibit cell growth and induce apoptosis	Bakrim et al. (2022).
Blood cancer	In vitro	U266 cells	β‐Sitosterol	Induced cytotoxicity and increased apoptotic populations	Sook et al. (2014).
Liver cancer	In vitro	HepG2 cells	β‐Sitosterol	Stimulating the Nrf2 and Nrf2/heme oxygenase‐1 pathways, decrease liver steatosis, inflammatory responses (IL‐1b and iNOS)	Abo‐Zaid et al. (2023).
Pancreatic cancer	In vitro	Acinar cells, Pancreatic ductal epithelial cells	β‐Sitosterol	NF‐kB suppressing effects, causing a G0/G1 cell cycle arrest, deactivating Akt/GSK‐3 action	Cioccoloni et al. (2022).

## 7 Bone Cancer

Phytosterols promote controlled multiplication of cancer cells and trigger a programmed cell death (apoptosis). This basic process plays a vital role in regulating the proliferation of tumors in different malignancies (Sicard et al. 2021). A major phytosterol, beta‐sitosterol, exhibits specifically pro‐apoptotic and anti‐proliferative properties on tumor cells. The fact that phytosterols can inhibit cell cycle development and promote apoptosis is of great importance in bone cancer, where cell proliferation is noticeably aggressive. Natural compounds are, in general, being studied to kill osteosarcoma cells because of their capacity to induce apoptosis and enhance autophagy. Phytosterols have the potential to disrupt essential signaling pathways that are commonly disrupted in cancer, which in turn amends cellular deformations that promote tumor growth and survival (Awari et al. 2024). As an example, some phytosterols can regulate important intracellular signaling pathways such as Akt/mTOR and JAK/STAT that are often over‐activated in many cancers such as ovarian and gastric cancers (Mir and Banik 2025). Such pathways are important to regulate tumor growth, and it is relevant to bone cancer, where certain signaling pathways, such as MAPK/ERK, PI3K/Akt/mTOR, and Wnt/beta‐catenin, are involved in its pathogenesis. Metastasis is usually associated with cancer progression and high mortality, particularly when it concerns aggressive cancers, such as osteosarcoma. The phytosterols also possess angiogenesis (growth of new blood vessels that serve tumors) and metastasis (moving of cancer cells to new places) inhibitory properties (Purnamayanti et al. 2022). In preclinical studies, it has been shown that phytosterols remain stable in the reduction of primary and metastatic tumor burden in a broad spectrum of cancer locations. They also reduce the indicators of metastasis and angiogenesis. This broad‐spectrum anti‐metastatic effect has been particularly helpful in bone cancer, including osteosarcoma that can be directly transferred to the lungs (Srivastava et al. 2025). Phytosterols are also highly anti‐inflammatory and antioxidative and can suppress cellular damage and regulate immunity, which leads to their overall anticancer effects. Such precautionary measures can assist in the development of a less optimal microenvironment of bone tumor proliferation and dissemination. Phytosterols also have potential benefits in reducing this resistance, which may increase the effectiveness of standard chemotherapeutic agents (Al‐Dhuayan et al. 2025). It is relevant to bone cancer, in which drug resistance formation is still an ongoing issue, and new agents are to be identified to make therapeutic responses better. Although a direct, comprehensive study on the clinical use of phytosterols, in particular in bone cancer, is the study that needs more attention, the developed general mechanism of anti‐cancer activity offers a solid scientific case in favor of its potential use. Moreover, natural compounds are undergoing active research on the treatment of osteosarcoma due to their prospective activity and minimal adverse effects, and cost (Awari et al. 2024). This type of interaction between beta‐sitosterol and bone cells, despite the presence of osteoporosis, implies direct interaction between beta‐sitosterol and bone biology, which can be developed into bone cancer delivery. The use of natural products as osteosarcoma therapies is a developing field of research. Traditional medicinal plants, with different natural compounds, such as phytosterols, are under investigation in their capacity to kill osteosarcoma cells in their capacity to disrupt pathways, like apoptosis induction and induced autophagy (Mir and Banik 2025). This may indicate that natural compounds, such as phytosterols, have potential as chemotherapeutic agents or sensitizers in drug combination therapies against bone cancer, but issues such as low bioavailability will have to be overcome. There are certain phytosterols such as beta‐sitosterol which have been known to react with bone cells. Indicatively, beta‐sitosterol has been indicated to exert a bone marrow mesenchymal stem cell osteogenic (bone‐forming) and adipogenic (fat‐forming) balance, even in osteoporosis. This is not specifically to do with the treatment of bone cancer but it shows a response to the bone microenvironment and may be of use in future bone cancer research (Awari et al. 2024).

## 8 Breast Cancer

Phytosterols are active agents in the prevention of the growth of breast cancer cells by inhibiting uncontrolled proliferation of cancerous cells and inducing programmed cell death (apoptosis) (Zhang, Yang, et al. 2025). The phytosterol beta‐sitosterol can cause G1 cell cycle arrest and cause mitochondrial membrane potential depolarization in human breast cancer MDA‐MB‐231 cells leading to apoptosis. It also demonstrates dose‐related anti‐proliferative effects on the human breast cancer MCF‐7 cells by inhibiting the cell cycle in G2/M phase (Asudas et al. 2025). The research shows that the antiproliferative effect of stigmasterol and beta‐sitosterol, the phytosterols, is associated with the morphological alterations including the chromatin condensation and nuclear fragmentation, and the deregulation of cell cycle (Andrés et al. 2025). Phytosterols induce apoptosis through the regulation of important signaling pathways. An example is beta‐sitosterol which is reported to stimulate Fas signaling of breast cancer cells. Besides, another phytosterol, ergosterol, was reported to increase the expression of tumor suppressor Foxo3 and its downstream signaling molecules, which result in apoptosis in human cells in the breast cancer cell line MDA‐MB‐231 (Asudas et al. 2025). Stigmasterol induces tumor cell apoptosis through control of the PI3K/Akt signaling pathway and the production of mitochondrial reactive oxygen species. Moreover, stigmasterol has a strong effect by down‐regulating anti‐apoptotic genes such as Bcl‐2 and BCL‐XL in breast cancer cell lines (Zhang, Yang, et al. 2025). In addition to in vitro findings, animal studies that induced rat mammary tumor models have reported that phytosterols decrease tumor volume and mass, which results in the increase of survival. One example is stigmasterol which was found to prevent tumor size in in vivo models of spontaneous breast tumors (Andrés et al. 2025).

The structural similarity of phytosterols to cholesterol means that it could affect the hormonal‐dependent growth of endocrine tumors, which is particularly applicable in estrogen receptor‐positive (ER+) breast cancers. The phytosterols have the potential to regulate the estrogen receptor signaling pathways, which tend to be major contributors to breast cancer development. The beta‐sitosterol and oleanolic acid have also been investigated in terms of their efficacy to inhibit the active estrogen synthesis enzyme human estrogenic 17beta‐hydroxysteroid dehydrogenase type‐1 (HSD17B1), which stimulates the growth of breast cancer. Molecular docking studies have demonstrated the interaction of phytosterols (e.g., beta‐sitosterol, campesterol, stigmasterol, and fucosterol) with the estrogen receptor. In addition to direct ER modulation, there also are phytoestrogens, which is the group to which some phytosterols belong, capable of anti‐cancer activity via aromatase inhibition, decreasing the generation of estrogen (Asudas et al. 2025). They are also able to bind to G protein‐coupled estrogen receptor 1 (GPER/GPR30), which provides an additional modulation option to estrogenic effects. Phytochemicals, such as phytosterols, may exhibit low or anti‐estrogenic activity with strong anti‐proliferative action, which provides nutritional or drug benefits in breast cancer therapy. One of the factors that cause morbidity and mortality in breast cancer is metastasis, the proliferation of cancer cells to other locations. The phytosterols have been shown to prevent angiogenesis (formation of new blood vessels that supply the tumors) as well as metastasis. The preclinical data are overwhelmingly clear that phytosterols, such as beta‐sitosterol, suppress both primary and metastatic tumor load in a broad spectrum of cancers, breast cancer among them (Zhang, Zhang, et al. 2025).

The phytosterol therapy in the breast and colorectal cancer models has constantly suppressed pAKT expression and certain metastasis indicators like alkaline phosphatase, matrix metalloproteins, epithelial to mesenchymal transition factors, and the colonization of the lung and brain. They also suppress indicators of angiogenesis such as vascular endothelial growth factor and CD31 (Mukherjee et al. 2025). Phytosterols, such as beta‐sitosterol and campesterol, decrease the in vitro metastatic capacity of human breast cancer cells by affecting tumor cell invasion, adhesion, and migration. An example of such is beta‐sitosterol, which can suppress the cell invasion of breast cancer cells by reducing the adhesive interaction of the tumor cell and the basement membrane. This interference with various steps of the metastatic cascade underscores their broad anti‐metastatic potential (Verma et al. 2025). Phytosterols can regulate key intracellular signaling pathways. Beta‐sitosterol and campesterol, e.g., have been demonstrated to influence the mevalonate and the MAP Kinase pathways in MDA‐MB‐231 human breast cancer cells, which are known to play a role in cell growth and apoptosis. Stigmasterol also regulates pathways like Akt/mTOR and JAK/STAT, which tend to lose their control in multiple cancers, including ovarian and gastric cancers among others (Li et al. 2025). The anticancer actions of beta‐sitosterol include stimulating apoptosis, arresting cell cycles, regulating oxidative stress, metabolic reprogramming, inhibiting invasion and metastasis, immunomodulation and inflammation, and overcoming drug resistance. Beta‐sitosterol is protective of oxidative damage as well. Certain phytosterones demonstrate considerable anti‐metastatic breast cancer cell effects on the MCF‐7 and anti‐inflammatory effects by inhibiting the formation of NO and TNF‐α (Monica et al. 2025). Oxidative stress and chronic inflammation are identified as factors in cancer progression and disease development. Phytosterols also have strong anti‐inflammatory/antioxidant effects that reduce cellular damage and regulate immune responses, which, in turn, are also linked to their total anticancer effects. They contribute to the establishment of a less conducive microenvironment for tumor growth and proliferation (Li et al. 2025).

## 9 Pancreatic Cancer

Phytosterols and specifically beta‐sitosterol have shown considerable effectiveness in the inhibition of pancreatic cancer cell growth and induction of programmed cell death (apoptosis). The use of beta‐sitosterol has been demonstrated to prevent the growth of pancreatic cancer cells. It does this by causing a G0/G1 cell cycle arrest and the cells cannot go through their division cycle (Cao et al. 2019). This mechanism assists in preventing the uncontrolled multiplication that is typical of pancreatic cancer. One of the most important points about the effect of beta‐sitosterol on pancreatic cancer is that it induces apoptosis. It is connected with up‐regulating the pro‐apoptotic proteins, such as Bax, and reducing the concentrations of anti‐apoptotic proteins, such as Bcl‐2. Through the modulation of these important regulators, beta‐sitosterol is a potent inducer of self‐destruction of pancreatic cancer cells. Pancreatic cancer often has an active nuclear factor kappa‐light‐chain‐enhancer of activated B cells pathway that helps to preserve cell survival, proliferate, and inflame (Ligeiro et al. 2020). The NF‐kB suppressing effects of beta‐sitosterol suggest that the compound plays a pivotal role in tumor growth suppression and may make these otherwise resistant cells vulnerable to the effects of other therapeutic agents. The extreme aggressiveness of pancreatic cancer usually necessitates the use of chemotherapy using gemcitabine as a very common drug. Nevertheless, resistance to drugs is a major threat. Phytosterols have the potential to overcome this resistance and improve treatment outcomes. Beta‐sitosterol is also discovered to react synergistically with gemcitabine in controlling anti‐pancreatic cancer (Adhimoolam et al. 2024).

This synergy effect means that the product is more powerful than the individual agents. This synergy is related to the property of beta‐sitosterol in the regulation of apoptosis and inhibition of epithelial‐mesenchymal transition, but inactivation of Akt/GSK‐3 activity. The Akt pathway is also prevalent in pancreatic cancer and plays a crucial role in the survival and growth of cells, and EMT is a mechanism whereby cancerous cells remodel such that they become more invasive and metastatic (Zhou et al. 2017). It suppresses this induction, whereby beta‐sitosterol stimulates the sensitivity of cancer cells to gemcitabine and reduces their motility. This suggests that phytosterols can be used as a complementary medicinal therapy to supplement the effectiveness of the existing chemotherapeutic regimens for the treatment of pancreatic cancer. Cancer in the pancreas is reputed to be highly metastatic, and this normally spreads to other body organs at earlier stages of the disease (Zhao and Liu 2020). Phytosterols have demonstrated the ability to lower primary and metastatic tumor burden in a variety of cancers, as well as act on metastasis and angiogenesis markers. Since pancreatic cancer is highly metastatic and thus new blood vessels (angiogenesis) are formed to feed the tumors and the proliferation of cancer cells later, the power of phytosterols to prevent new blood vessels (angiogenesis) formation and to inhibit cancer cell proliferation could be of crucial importance in the regulation of disease progression. Phytosterols disrupt a variety of cell signaling pathways that promote cancer development, such as cell cycle control, survival, invasion, and inflammation (Khan et al. 2023). These extensive modulatory interactions can be useful in addressing the multifaceted molecular environment of pancreatic cancer in which several pathways are frequently altered.

## 10 Prostate Cancer

Phytosterols have been shown to suppress the proliferation of tumor cells in prostate cancer and actively induce programmed cell death (apoptosis), which causes the self‐destruction of cancer cells. Phytosterols, such as beta‐sitosterol, cause cell cycle arrest in the cancer cells of the prostate, thus inhibiting their uncontrolled proliferation (Henriksbo et al. 2019). The mechanism makes the cancer cells stop their division stages, which inhibits tumor progression. Phytosterols prefer the apoptotic process, which is useful in eliminating cancer cells. A single major phytosterol, beta‐sitosterol, has been particularly put forward to have a pro‐apoptotic effect on prostate epithelial cells. It has been found that the antiproliferative activity of phytosterols such as beta‐sitosterol is linked to morphological alterations of chromatin condensation and nuclear disintegration. Among the simplest elements of the anticancer effect of phytosterols, one can distinguish its capacity to induce the death of tumor cells. Some studies found the phytosterols to relieve various types of cancer, including prostate cancer, by retarding the growth of cancerous cells (Batista et al. 2021). This is one of the potential contributions of this net effect to the treatment of prostate cancer. Besides the direct influence of cancer cell survival, angiogenesis, or the formation of new blood vessels, has also been found to be affected by phytosterols, so that tumors can get nutrients and oxygen. Phytosterols can block this process, which starves prostate tumors and slows their growth and spread. The angiogenic mechanism that is required to sustain and develop tumors is interfered with by phytosterols. Whereas direct evidence of prostate cancer is yet to be defined, in other models, beta‐sitosterol has been shown to modulate angiogenesis, including by blocking the VEGF signaling in rheumatoid synovial angiogenesis and by inhibiting angiogenesis signatures in a rheumatoid carcinogenesis model. The same could be said about prostate cancer, considering that such an effect can be made through the general ability of phytosterols to prevent angiogenesis (Wang et al. 2022).

Phytosterols can also stop the growth of prostate tumors and slow down their progression by depriving them of blood. This mechanism is critical for controlling the growth of cancerous aggressions. The importance of androgen receptor signaling has emerged as one of the mechanisms involved in the development and progression of prostate cancer, which is usually hormone‐dependent (Cioccoloni et al. 2022). Phytosterols are able to participate in this hormonal mechanism. The interest has been drawn to phytosterols, such as beta‐sitosterol, stigmasterol, and campesterol, with their 5α reductase enzyme‐inhibitory effects. The enzyme is involved in the conversion of testosterone to dihydrotestosterone, which is a potent androgen that drives prostate enlargement and is crucial in the growth and progression of benign prostatic hyperplasia and prostate cancer. Phytosterols have the capacity to reduce the amounts of androgenic signal, which drives the expansion of prostate tumors (Kang et al. 2021). Beta‐sitosterol is also reported to prevent the binding of DHT to the androgen receptor, which also disrupts the hormonal signaling required to allow prostate cancer to grow. Phytosterols have the potential to work on the growth of hormone‐dependent endocrine tumors in general. This broad inducing impact on hormonal signaling is particularly relevant to prostate cancer, as a significant proportion of these types of cancer rely on androgen signaling to thrive and proliferate. In addition to all these specific mechanisms, phytosterols also exhibit anti‐inflammatory and antioxidant properties, which are also relevant to their overall anticancer effects to minimize cell damage and immunological responses in the prostate microenvironment (Hao et al. 2022). The multi‐targeted mechanism of action of phytosterols predisposes them to be a promising research area in the future of prostate cancer prevention and treatment.

## 11 Colon Cancer

Phytosterols have a direct anti‐proliferative effect on colon cancer cell growth and actively induce apoptosis, resulting in the death of cancerous cells. It was demonstrated that phytosterols, such as beta‐sitosterol, campesterol, and stigmasterol, can significantly lower the viability of colon cancer cells (e.g., Caco‐2 cells) at concentrations that are similar to human colon concentrations of these compounds (Cilla et al. 2015). In particular, stigmasterol was said to have a strong antiproliferative effect. It is also suggested by the rodent models that the effect of phytosterols in preventing the process of colon carcinogenesis is possible by disrupting cell cycle progression. This means they can prevent the unregulated growth of cancerous colon cells by preventing their advancement into definite stages of the cell cycle. The apoptosis (programmed death) of colon cancer cells is brought about by phytosterols (Alvarez‐Sala et al. 2019). One would include beta‐sitosterol, which has been proven to induce apoptosis of human colon cancer cells by activating pro‐apoptotic proteins, including Bax and caspases, which are enzymes participating in the apoptotic process. This is among the primary ways by which this pro‐apoptotic mechanism of phytosterols combats colon cancer. Beta‐sitosterol has been shown to have chemopreventive effects in experimental colon cancer models via induction of apoptotic cell death and effects on significant cellular markers, including beta‐catenin and PCNA (Nandi et al. 2024). Phytosterols are also effective in inhibiting the development of tumors and the growth of colon cancerous cells. It is preclinically demonstrated that phytosterols can reduce the tumor burden in various cancers, such as colorectal cancer. Their direct inhibitory activity against tumor growth is another characteristic significant of their anticancer potential (Naeem et al. 2022).

The phytosterols are also involved in cancer prevention, which inhibits metastasis. The phytosterol therapy in the models of colorectal and breast cancer had always reduced the concentrations of pAKT and several of the metastasis‐linked biomarkers, such as alkaline phosphatase, metalloproteases, and epithelial to mesenchymal transcription factors. It means that phytosterols can interfere with the processes of colon cancer cells' invasion into the surrounding tissue and their movement to other organs that are located far away (Shahzad et al. 2017). Phytosterol antioxidant and anti‐inflammatory properties also come in handy, and this applies more to the colon and cancer prevention. Stigmasterol is a significant phytosterol, and it was established that it can cause an overproduction of reactive oxygen species in colon cancer cells, which can also contribute to their cytotoxic activity (Bakrim et al. 2022). Even though ROS can be detrimental, it can induce apoptosis in case it occurs when the increase of cancer cells is controlled. Phytosterols are anti‐inflammatory, and they can prove useful in the given conditions like colitis, which is a predisposing factor of colon cancer. Phytosterols can also prevent the occurrence of the disease by reducing inflammation of the colon (Rocha et al. 2016).

Although further interventional research is required to completely test the effectiveness of various types and forms of phytosterols in cancer prevention, current evidence on the subject by in vitro and in vivo research clearly indicates their potential in the fight against colon cancer. The fact that they are localized in the colon because of their low absorption makes them especially applicable in targeting this kind of cancer (Banerjee et al. 2017).

## 12 Oral Cancer

The growth factors in normal cells interact with some receptors to stimulate cell growth and differentiation. However, cancer cells develop autonomous and unstructured properties owing to the loss of control of growth signals. They have established that the overexpression of epidermal growth factor receptor (EGFR) is closely associated with a high clinical stage of OSCC, low survival rate, and recurrence rate. One of the most frequently altered signaling pathways in the squamous cell carcinoma of the head and neck is the EGFR/PI3K/Akt cascade. PI3K signaling is initiated by the binding between EGFR and its ligands EGF or transforming growth factor‐alpha (TGF‐alpha), and after that by stimulating the generation of phosphatidylinositol 3,4,5 trisphosphate, resulting in the activation of Akt (Alzawi et al. 2022). Then, Akt activates the antiapoptotic signaling mediated by NF‐kB and suppresses the proapoptotic targets transcription via FOXO to promote cell survival. Moreover, cyclin D1 also regulates the cell cycle between G1 and S. Overexpression of cyclin D1 causes a shortening in G1 phase, resulting in abnormal proliferation of cells, which may eventually promote the occurrence of other genetic damages. It has been demonstrated that the degree of differentiation, the presence of metastasis in lymph nodes, and the poor prognosis of OSCC are the causes of contraindication of cyclin D1 expression (Ramos‐Garcia et al. 2018). Ras is considered to be one of the most prevalent mutant oncogenes of oral cancer. The point mutations of the Ras gene can lead to the permanent activation of the Ras protein and cause permanent cell growth. In addition, the signal transducer and activator of transcription protein family is also highly relevant in the development of oral cancer. In one of the studies, the same authors revealed that the presence of STAT3 promotes OSCC cell migration, invasion, epithelial‐mesenchymal transition (EMT), and aerobic glycolysis by inhibiting FOXO1 transcription. In addition, one of the studies demonstrated that low OSCC prognosis is linked to the expression of phosphorylated STAT3 (Zheng et al. 2019).

## 13 Liver Cancer

Phytosterols (beta‐sitosterol) can modulate the concentration of ROS in hepatocancer cells. Although beta‐sitosterol has been reported to induce ROS‐mediated apoptosis in human hepatocellular carcinoma cells, a review study has revealed that beta‐sitosterol can bidirectionally regulate oxidative stress, i.e., can both stimulate and scavenge ROS, depending on cellular context, which is ultimately involved in its anticancer effects (Ditty and Ezhilarasan 2021). The apoptosis of tumor cells is also induced by stigmasterol, which regulates the production of mitochondrial reactive oxygen species. Phytosterols can supplement the natural antioxidant defense mechanism of the body. They facilitate the activity of important antioxidant enzymes, including superoxide dismutase, catalase, and glutathione. These enzymes play critical roles in countering adverse free radicals as well as countering oxidative damage to liver cells (Li et al. 2015). Phytosterols may counter lipid peroxidation, which is the free radicals destroying lipids and thus results in dysfunctional cells and the development of cancer. This inhibition can be performed by stimulating the Nrf2 and Nrf2/heme oxygenase‐1 pathways that play a major role in cellular protection against oxidative stress. In addition to direct anticancer effects, phytosterols have hepatoprotective effects in general. Beta‐sitosterol is also reported to decrease liver steatosis, inflammatory responses (IL‐1b and iNOS), and liver endoplasmic reticulum stress in high‐fat diet‐fed rats, suggesting its potential to keep the liver healthy and fight conditions that may lead to cancer development (Tanasa et al. 2025). Beta‐sitosterol has been shown to cause reactive oxygen species‐mediated apoptosis in human hepatocellular carcinoma cell lines. Beta‐sitosterol, which was extracted from *Indigofera zollingeriana* extract, showed cytotoxicity activity against HepG2 and Huh7 liver cancer cells in an experimental environment. HepG2 cells were reported to have a half‐maximal inhibitory concentration of beta‐sitosterol of 6.85 ± 0.61, and Huh7 cells of 8.71 ± 0.21 μg/mL (Vo et al. 2020).

It is a sign of a dose‐dependent anticancer effect. Beta‐sitosterol has been recognized to regulate oxidative stress among other processes, including the induction of apoptosis and cell cycle arrest, which has been credited with the anticancer effects of beta‐sitosterol in a large number of cancers, including liver cancer. Stigmasterol has already been determined to have anti‐oxidizing properties. It has been found that stigmasterol has anti‐tumor activity in liver cancer both in vivo and in vitro. It does so by causing apoptosis and inhibiting proliferation, metastases as well as invasion of tumor cells. Stigmasterol causes tumor cell apoptosis mechanically by changing the PI3K/Akt signal and mitochondrial reactive oxygen species (Zhang et al. 2022).

## 14 Gastric Cancer

Phytosterols also show anticancer activity against gastric (stomach) cancer, and their antioxidant capacity is suspected to be the source of this activity. Although thorough and detailed studies on the specific antioxidant mechanisms using exact doses in gastric cancer remain under exploration, there is current evidence on their protective effect. Epidemiological research indicates a correlation between phytosterol consumption in the diet and low risk of stomach cancer. A case–control study in Uruguay has concluded that there was a strong negative association between total phytosterol intake and stomach cancer (odds ratio = 0.33, 95% confidence interval = 0.17–0.65 in the highest tertile). Part of this protective effect is due to their capacity to neutralize oxidative damage (Rudrapal et al. 2022). Phytosterols have antioxidant activity and can eliminate free radicals and decrease oxidative stress in cells. This capability is crucial to prevent cellular damage that can initiate and promote the development of cancer even in the stomach. Phytosterols inhibit the growth of stomach cancerous cells. This includes a direct effect on cell viability and proliferation. Although the direct correlation to the antioxidant system is not always quantitatively determined in such research, depletion of oxidative stress may help in these inhibitory actions by providing a less conducive environment to the growth of cancer cells. Phytosterols can affect a number of cellular activities that play a role in cancer. The well‐known phytosterol stigmasterol has exhibited anti‐tumor effects in gastric cancer in vivo and in vitro (Alnoor et al. 2024).

It is capable of inhibiting the Akt/mTOR‐signaling pathway in gastric cancer cells, which is commonly dysregulated in cancer and in some cases is affected by oxidative stress. A major predisposing factor to gastric cancer is chronic inflammation. Phytosterols have anti‐inflammatory effects, and this may indirectly impact anticancer actions by alleviating the inflammatory burden that may cause increased oxidative stress and cell damage in the stomach lining. It has been found that stigmasterol is anti‐tumor against gastric cancer. A physical experiment showed that stigmasterol was able to inhibit tumor growth in a gastric cancer xenograft model (Zhao et al. 2021). Although the mechanisms such as apoptosis and autophagy were the main focus of this research, stigmasterol is also characterized by its anti‐oxidation effect. The cell growth and apoptosis of SGC‐7901 human stomach cancer cells have been reported to be inhibited and induced by beta‐sitosterol. It has a number of anticancer actions in stomach cancer and is involved in augmenting apoptosis, cell cycle stoppage, and control of immunity and inflammation. It is also discovered that beta‐sitosterol may bi‐directionally regulate the oxidative stress, i.e., cause and scavenge ROS depending on the conditions of the cell, and the action ultimately contributes to its anticancer action. Campesterol along with beta‐sitosterol and stigmasterol belongs to the total dietary phytosterol concentrations that are associated with a reduction in the risk of stomach cancer (Gupta 2020).

## 15 Kidney Cancer

The anticancer activities of phytosterols are usually multifaceted, due to the ability of these substances to inhibit cell growth, induce apoptosis (programmed cell death), and suppress angiogenesis (the growth of new blood vessels feeding the tumors). A high phytosterol, beta‐sitosterol, has been examined in a rat model of experimental renal carcinogenesis. The use of beta‐sitosterol orally was found to greatly reverse the expression of angiogenesis, proliferation, and apoptotic markers, implying that it is protective against renal cancer. Phytosterols are generally antioxidants, and this is one of the reasons they protect cells against damage (Poudel et al. 2023). Phytosterols can be used to counteract bad free radicals, hence relieving oxidative stress that may cause damage to cells and lead to cancer. Phytosterols can affect a number of cell activities. As an example, beta‐sitosterol has been found to provide defense against oxidative damage. Phytosterols can stimulate antioxidant defense mechanisms in the body. A recent study suggested that dietary phytosterol could prevent renal fibrosis by between stigmasterol and beta‐sitosterol stimulating mitophagy, in which damaged mitochondria are eliminated, which can eliminate oxidative stress (Yang et al. 2025).

This suggests a potential but significant role in maintaining the health of the kidneys by controlling oxidative stress. Beta‐sitosterol (20 mg/kg body weight) is protective as it reverses the angiogenesis, proliferation, and apoptosis indicators in an experimental rat model of renal carcinogenesis when delivered orally. Another study involving high‐fructose diet‐fed rats found that oral administration of 20 mg/kg body mass of beta‐sitosterol inhibited the occurrence of oxidative stress and hepatorenal derangements, which means that beta‐sitosterol has the ability to prevent the damage and lipid peroxidation caused by ROS in the liver and kidneys. Oxidative DNA damage has also been used to determine the effects of beta‐sitosterol on antigenotoxic and anticancer action. Beta‐sitosterol is confirmed to be able to offer protection against oxidative damage (Kaur et al. 2023).

Beta‐sitosterol is known to have a variety of anticancer effects in many types of cancer and the possibility of regulating the oxidative stress pathway by bidirectionally regulating ROS. Stigmasterol is recognized because of its anti‐oxidizing effects. It is also able to stimulate SOD and CAT activity, as well as reducing ROS generation. The fact that renal fibrosis reduction occurs through its activating effect on mitophagy further demonstrates its indirect contribution towards kidney health as an antioxidant. A chronic kidney disease mouse study involved dietary supplements of stigmasterol and beta‐sitosterol. High‐dose phytosterols indicated that phytosterols were more effective in inhibiting renal fibrosis and oxidative stress, suggesting that phytosterols provided a dose‐dependent protection effect on kidney disease (Adamantidi et al. 2024).

## 16 Blood Cancer

The anticancer effects of phytosterols are diverse and can be observed in a wide range of malignancies, such as alleviation of some blood cancers. Phytosterols such as beta‐sitosterol have been shown to induce apoptosis (programmed cell death) of multiple cancer cells, and include those that are pertinent to blood cancers (Alvarez‐Sala et al. 2019). Beta‐sitosterol induces apoptosis in multiple myeloma U266 cells. It is accompanied by mitochondrial potential reduction and caspase activation. The phytosterols are also able to prevent the growth of cancer cells by halting the cell cycle, thus avoiding uncontrolled growth. The impact of phytosterols on the level of ROS is the key to their antioxidant activity. Oxidative stress is bidirectionally regulated by beta‐sitosterol, i.e., it can induce and scavenge ROS, depending on the cellular setting (Abo‐Zaid et al. 2023). This property accounts for its anticancer potential as it can induce the ROS‐mediated cancer cell death or protect healthy cells against oxidative stress. ROS have been indicated to mediate beta‐sitosterol‐induced apoptosis of multiple myeloma cells. Antitumor effects may also be caused by phytosterols due to enhanced recognition of cancer cells by the immune system. There is overwhelming preclinical evidence that beta‐sitosterol has various anticancer activities against a range of cancers, such as leukemia and multiple myeloma (Dwivedi et al. 2024). Failure to identify the molecular action of beta‐sitosterol in multiple myeloma U266 cells, this compound caused cytotoxicity and amplified apoptotic subpopulations, which was mediated by reactive oxygen species‐dependent stimulation of AMP‐activated protein kinase and c‐Jun N‐terminal kinase pathways. The paper has reported a dose‐related effect on cytotoxicity, but direct numerical doses related to antioxidant indicators of blood cancer were not specified in the passage. Beta‐sitosterol can prevent and treat cancer through the following mechanisms: increasing apoptosis, cell cycle arrest, and bidirectional regulation of oxidative stress (Bao et al. 2022).

## 17 Brain Cancer

Oxidative stress is important in the pathogenesis and evolution of different cancers, such as brain cancer. Oxidative damage is especially prone to brain damage because of its high metabolic rate and lipid concentrations. Antioxidant properties of phytosterols are well‐known, and they have attracted attention because of their possible therapeutic benefits in terms of eliminating oxidative stress and preventing the growth of brain tumors. Phytosterols have a direct ability to neutralize harmful free radicals, which lessen oxidative stress that may destroy DNA, proteins, and lipids, all of which play a role in carcinogenesis (Muscolo et al. 2024). Phytosterols may also reduce the levels of ROS, prevent the oxidative injury of brain cells, and avoid pathways that distinguish the growth of the tumor. Phytosterols have the potential to stimulate endogenous antioxidant enzyme activity of the brain cells, including superoxide dismutase, catalase, and glutathione peroxidase. Such enzymes play an essential role in the detoxification of free radicals and the cellular redox balance. Chronic inflammation is usually associated with augmented oxidative stress in the tumor microenvironment. Phytosterols have anti‐inflammatory effects that may indirectly help decrease oxidative load and furnish a less conducive environment to brain tumor development (Jie et al. 2022). Phytosterols are able to induce programmed cell death (apoptosis) and cell cycle arrest of cancer cells, including cancer cells in brain tumors. This process may at times be associated with the regulation of oxidative stress, in which a regulated ROS increase may result in cancer cell death, and decreased overall oxidative stress may safeguard healthy cells (Arfin et al. 2021).

Beta‐sitosterol is a highly promising anticancer agent, including against brain tumor cells, and among the most common phytosterols. It is able to affect ROS levels and play a role in inducing apoptosis in cancerous cells. Although the effect of antioxidants on brain cancer is not always directly measured, the fact is that it can be bidirectionally regulated in response to oxidative stress is a common action of most types of cancer. Research has suggested that beta‐sitosterol can promote cytotoxicity and apoptosis of some human brain tumor cell types (a form of brain cancer) via mechanisms that involve the regulation of oxidative stress, though specific antioxidant dosages needed in this particular activity frequently necessitate more focused studies (Adhimoolam et al. 2024).

## 18 Cervical Cancer

In cervical cancer, there has been an increase in the level of oxidative stress in association with the disease, and a low level of antioxidants has been linked to heightened production of free radicals, lipid peroxidation, DNA damage, and protein damage, all of which play a role in malignant transformation. Thus, one of the potential approaches to cervical cancer prevention and treatment is the targeting of oxidative stress (Silva et al. 2018).

Phytosterols play a role in anticancer by having multi‐targeted mechanisms. The inbuilt antioxidant properties of phytosterols enable them to neutralize the adverse free radicals directly. A multi‐directional process, oxidative stress is under the control of the most popular phytosterol, which is beta‐sitosterol (Wang and Mao 2024). It uses that it is able not only to scavenge excess ROS to prevent cell damage but also, in some settings in cancer, induces a reactive amount of ROS, which leads to cell death in cancer cells. Such dual capability makes phytosterols potentially possess the capacity to restore cellular redox balance, which is the key to cancer prevention and management. Phytosterols can trigger the activation of major endogenous antioxidant enzymes, which are significant components of the cellular response to oxidative stress (Vezza et al. 2020). Superoxide Dismutase converts the superoxide radicals to oxygen and hydrogen peroxide that is less harmful. Catalase converts hydrogen peroxide to water and oxygen. Glutathione itself neutralizes the effect of the free radicals and is involved in the processes of detoxification. The activation of these enzymes aids in alleviating the burden of oxidative damage in the development of cervical cancer (Preci et al. 2021). NF‐E2‐FGF2 is a principal controller of the antioxidant response in cells. Activation of Nrf2 induces the expression of many genes that encode antioxidant enzymes and cytoprotective proteins. Generally, phytosterols have been reported to induce the Nrf2 and Nrf2/heme oxygenase‐1 signal transduction, which inhibited lipid peroxidation and increased cellular resistance to oxidative stress (Mansouri et al. 2022).

Although research directly implicating phytosterol‐mediated Nrf2 in cervical cancer is not described in detail, this broad mechanism is indicative of a potential role. They are processes that are usually closely connected with oxidative stress. Although the specific molecular connection between these direct effects on cells of the cervix and the antioxidant potential of phytosterols is yet to be determined, through the modulation of pathways such as Ras/ERK and PI3K/Akt, the cellular redox state may be indirectly and positively influenced to induce cell death or inhibition of growth in cancer cells (Rezatabar et al. 2019).

## 19 Uterine Cancer

Numerous factors affect uterine cancer, which encompasses endometrial cancer and cervical cancer. Phytosterols are phytonutrients that are plant‐based compounds with a variety of anticancer effects. Malignant transformation may be mediated by oxidative stress, which is an imbalance between reactive oxygen species formation and antioxidant defense. Anticancer effects of phytosterol, especially beta‐sitosterol have been demonstrated on cervical cancer. Phytosterols contribute to anticancer effects through complex, multi‐targeted mechanisms (Mubeen et al. 2025). It is found that Beta‐sitosterol is bidirectional in the control of oxidative stress. This implies that it may help to reduce excessive ROS as well as cause‐controlled amounts of ROS, resulting in cell death in cancerous cells. Stigmasterol induces apoptosis of ovarian cancer cells through ROS generation, and campesterol has been reported to regulate oxidative stress, which results in the generation of ROS in ovarian cells. Stigmasterol has anti‐proliferative and pro‐apoptotic actions in diverse cancers including ovarian cancer and these effects are linked to the control of mitochondrial reactive oxygen species. Nrf2 is a vital element of cell defense pathway (Bakrim et al. 2022). One NRF2 inhibitor (stigmasterol) that increases the sensitivity of endometrial cancer cells to chemotherapy by reducing the levels of Nrf2 protein was discovered. Phytosterols modulate cell growth, cell survival, and cell death cellular mechanisms, and have anti‐proliferative properties and induce apoptosis in cancer cells. It was shown that beta‐sitosterol can affect cervical cancer protection by inhibiting cell proliferation. The processes help to stop the proliferation that is unregulated in the case of cancer (Enane et al. 2018).

## 20 Thyroid Cancer

Phytosterols are effective antioxidants that counteract free radicals and reactive oxygen species (ROS), which act in cancer cell proliferation and metastasis. ROS plays a major role in the emergence and progression of most cancers, such as thyroid cancer. Phytosterols also help in the reduction of oxidative stress to guarantee cellular redox, and therefore prevent the destruction of DNA, cell mutation, and cancer. Phytosterols also trigger the work of antioxidant enzymes like superoxide dismutases, catalase, and glutathione peroxidases (GPx), which counter the ROS (Cordiano et al. 2025). The phytosterols reduce the inflammation by inhibiting the production of proinflammatory cytokines and transcription factors like the nuclear factor kappa‐light‐chain‐enhancer of activated B cells (NF‐kB). They block the expression of pro‐inflammatory molecules (e.g., TNF‐α and IL‐6) contributing to the tumor microenvironment, which makes cancer cells less susceptible to apoptosis. Phytosterols also inhibit the COX and LOX enzymes that are linked to the production of the pro‐inflammatory mediators like prostaglandins and leukotrienes (Altomare et al. 2025). These enzymes are the ones that are involved in long‐term inflammation, which is normally experienced in cancerous tissues, as is the case with thyroid, which are blocked by the phytosterols. The apoptosis (programmed cell death) in thyroid cancerous cells by the phytosterols is shown by the expression of the significant apoptotic proteins, caspases, and the Bcl‐2 family proteins. Phytosterols can trigger the mitochondrial apoptotic pathway that increases the mitochondrial membrane permeability, releases cytochrome c, and causes the cleavage of caspases. Moreover, the phytosterols cause the cell cycle arrest, particularly at G1/S phase, by regulating the cyclin‐dependent kinases (CDKs) and cyclins. This hindrance of cell division prevents an uncontrolled increase in the number of cells—an event that happens to cancer cells (Talib et al. 2022). Phytosterols inhibit the PI3K/Akt pathway that is normally over‐regulated towards cancer, including thyroid cancer. Blocking this pathway, phytosterols prevent the survival and proliferation of cancer cells.

Phytosterols also inhibit the MAPK/ERK signaling pathway, which regulates cellular functions including growth, differentiation, and apoptosis, which additionally evidences the anticancer potential of phytosterols (Tornese et al. 2024).

## 21 Skin Cancer

Ultraviolet (UV) radiation and environmental factors are responsible for skin cancer and result in heightened oxidative stress and damage to DNA as well as inflammation. Phytosterols have a great potential to be used as a therapeutic agent with their strong antioxidant effects in reducing the impact of skin cancer. One of the causes of skin cancer, especially UV‐induced carcinogenesis, is oxidative stress. The high antioxidant properties of phytosterols have great potential to be utilized as a therapeutic agent in mitigating the effects of skin cancer. Oxidative stress is among the factors that result in skin cancer and carcinogenesis induced by UV. The phytosterols are antioxidants that counter free radicals and fend off oxidative stress in the skin cells. Phytosterols like stigmasterol and β‐sitosterol have been shown directly to counter free radicals like superoxide anion and hydroxyl radical, which are byproducts of UV radiation (Taylor et al. 2022). Inflammation plays a significant part in the pathogenesis and in the pathogenesis of skin cancer, particularly UV‐induced skin carcinogenesis. Phytosterols possess anti‐inflammatory properties by regulating a number of inflammatory mediators. Phytosterols inhibit the production of inflammatory cytokines such as TNF‐α, IL‐1, and IL‐6, which contribute to the occurrence of skin cancer. They also prevent the activation of NF‐kB, which is a critical transcription factor in the expression of these cytokines. The expression of cyclooxygenase‐2 (COX‐2) and inducible nitric oxide synthase (iNOS), which are hyperexpressed in cancerous cells of the skin, has been suppressed by phytosterols and leads to inflammation and tumorigenesis. This is an anti‐inflammatory effect that contributes to the reduction of the chronic inflammatory state of the tumor microenvironment and, consequently, inhibits the progression of cancer (Liu et al. 2023). Phytosterols stimulate skin cancer cell apoptosis by preventing intrinsic and extrinsic apoptotic pathways. They distort the expression of pro‐apoptotic proteins, including Bax, and inhibit the expression of the anti‐apoptotic proteins, including Bcl‐2. This triggers the caspases to become activated, which results in an apoptotic cascade that ultimately kills the cancerous cell. The phytosterols lead to cell cycle arrest, particularly in the G1/S phase, due to a balance in the cyclin‐dependent kinases (CDKs) and cyclins (Taylor et al. 2022).

This prevents the growth of cancer cells in the cell cycle and uncontrolled division. One of the major causes of UV radiation is skin cancer, which is typified by melanoma, basal cell carcinoma, and squamous cell carcinoma. Phytosterols have been shown to protect against UV damage to the skin. Phytosterols reduce UV‐induced DNA damage by scavenging free radicals and repairing the offshoots of the DNA lesions. They also increase the action of the DNA repair proteins like PARP (poly (ADP‐ribose) polymerase), which helps in repairing the UV damage of the DNA. Phytosterol regulates the synthesis of melanin, which can have certain protective effects against UV rays. Phytosterols also make exposure to UV radiation less harmful since they are capable of regulating the production of melanin in the skin cells (Zhao et al. 2025).

## 22 Bladder Cancer

Genetic mutations and environmental exposures are the major causes of bladder cancer, including smoking, which causes oxidative stress and chronic inflammation. Plant‐based compounds have shown a potential therapeutic effect on the prevention of bladder cancer due to their antioxidant, anti‐inflammatory, and anticancer properties, including phytosterols. Phytosterols have a strong effect on bladder cancer cells through several mechanisms, which provide an alternative or an add‐on to conventional therapies. Phytosterols, including β‐sitosterol, neutralize reactive oxygen species (ROS), which increase in excess during bladder cancer development, particularly on exposure to carcinogens such as cigarette smoke. Phytosterols lessen cellular destruction by clearing these free radicals and maintain normal cellular activity, inhibiting the onset of cancer (Khan et al. 2023).

Phytosterols increase the activities of the natural antioxidant enzymes like superoxide dismutase (SOD), catalase, and glutathione peroxidase (GPx) of the bladder cancer cells. These enzymes neutralize ROS and restore the cellular redox state and thus prevent oxidative DNA damage and mutations that can cause cancer progression. The NF‐kB signaling pathway is among the most important regulators of inflammation in bladder cancer, and it induces the pro‐inflammatory cytokines that promote tumor development. NF‐kB activation by phytosterols is prevented, and hence the synthesis of inflammatory agents like TNF‐α and IL‐6 that would otherwise favor survivability and apoptotic resistance of cancer cells is blocked. COX‐2 and inducible nitric oxide synthase (iNOS) are also expressed in bladder cancer and contribute to the development of chronic inflammation and cancer. Phytosterols also inhibit COX‐2 and iNOS, which cause a reduction in the production of inflammatory mediators, including prostaglandins and nitric oxide, that mediate the tumorigenesis of bladder cancer. Phytosterols trigger mitochondrial dysfunction through the regulation of pro‐apoptotic proteins like Bax and p53, leading to the release of cytochrome c and caspase (Maaz et al. 2025).

This cascade is eventually followed by cancer cell death. Additionally, phytosterols stimulate cell surface death receptors (extrinsic pathway) that also stimulate apoptosis. The Bcl‐2 type proteins are locally specific apoptotic regulators, allowing the coordination of the anti‐apoptotic and pro‐apoptotic signals. Phytosterols trigger apoptosis in bladder cancer by increasing the expression of the pro‐apoptotic protein, such as Bax, and decreasing Bcl‐2. This is the expression change in favor of cancer cell death in Bcl‐2 family proteins. Phytosterols inhibit tumor cell uncontrolled growth by blocking cell cycle progression by regulating the actions of cyclin‐dependent kinases (CDKs) and cyclins on them and inhibit the abnormal or unregulated proliferation of tumor cells. Phytosterols enhance the transcription of tumor suppressor‐like p53 which is a vital protein in cell cycle and DNA repair. Phytosterols cause cell cycle arrest and provide time during which DNA could repair itself by activating p53. It is also the case that phytosterols inhibit the actions of the protein Cyclin D1 that facilitates the growth of cancerous cells in the bladder. Phytosterols reduce the activity of the matrix metalloproteinases (MMP), which degrade the extracellular matrix and allow the cancer cell to enter tissues around it (Talib et al. 2022). Phytosterols inhibit the invasion of bladder cancer cells since they inhibit the expression of MMPs, hence preventing the cell metastasis. In EMT, the process involves having the epithelial cells develop into mesenchymal cells, making them gain more ability to migrate and invade other tissues. Phytosterols have been reported to prevent EMT in the cancer cells of the bladder which suppress their metastatic ability and limit the growth of the tumor (Maaz et al. 2025). Table 2 explores the In vivo/In vitro studies of Anticancer Properties of Phytosterols.

## 23 Conclusion

Phytosterols are a structurally diverse group of plant sterols which have a dramatic anticancer action with multi‐targeted molecular pathways. Modern mechanistic investigations indicate that stigmasterol and campesterol tune major oncogenic signaling cascades such as PI3K/Akt/mTOR, NF‐kB, Wnt/–catenin, and caspase‐activated apoptotic pathways and hence prevent tumor cell proliferation, angiogenesis, and metastasis. Cytotoxic and chemo‐sensitizing activities of phytosterols have been verified in a preclinical and limited clinical study in a variety of malignancies including breast, prostate, colorectal, hepatic, and pancreatic cancer. Dose‐effect has been demonstrated by experimental dosage (25–400 mg/kg in animals, 1–4 g/day in humans), showing dose‐effect without serious systemic toxicity. Significantly, the recent improvements in nanocarrier‐mediated delivery systems, such as liposomal, micellar, and polymeric nanoparticles, have significantly increased the bioavailability and pharmacokinetic stability of these lipophilic compounds, which have increased their translational viability. Regardless of such encouraging findings, there is still a lack of clinical validation with methodological heterogeneity, a lack of standardization of phytosterols, and long‐term human‐based data. The first approach that needs to be considered in future research is pharmacokinetic modeling, integration of multi‐omic biomarkers, and randomized, placebo‐controlled clinical trials using standardized formulations. The body of evidence has collectively placed phytosterols as effective, safe, and multi‐mechanistic bioactive agents with the prospect of being used as adjuvant agents in precision oncology. Standard dosing structures and explanation of individual patient responses will be important steps in the process of their clinical adoption as nutraceutical or therapeutic adjuncts in cancer therapy.

## Author Contributions

**Muhammad Shahbaz:** conceptualization, writing – original draft. **Ushna Momal:** conceptualization, writing – original draft. **Asfa Perween:** writing – original draft, investigation. **Hammad Naeem:** writing – review and editing, validation, visualization. **Muzzamal Hussain:** writing – review and editing, supervision. **Muhammad Imran:** resources, methodology. **Gamal A. Mohamed:** data curation, investigation. **Sabrin R. M. Ibrahim:** supervision, writing – review and editing. **Suliman A. Alsagaby:** investigation, data curation, software. **Waleed Al Abdulmonem:** writing – original draft. **Entessar Al Jbawi:** supervision, validation, visualization. **Mohamed A. Abdelgawad:** data curation, investigation, resources. **Samy Selim:** writing – review and editing. **Soad K. Al Jaouni:** writing – review and editing. **Hagar M. Mohamed:** writing – review and editing, data curation.

## Funding

The authors have nothing to report.

## Conflicts of Interest

The authors declare no conflicts of interest.

## Data Availability

The data that support the findings of this study are available from the corresponding author upon reasonable request.
